# RibMR – A Mixed Reality Visualization System for Rib Fracture Localization in Surgical Stabilization of Rib Fractures: Phantom, Preclinical, and Clinical Studies

**DOI:** 10.1007/s10278-024-01332-2

**Published:** 2024-12-20

**Authors:** Hoijoon Jung, Jineel Raythatha, Alireza Moghadam, Ge Jin, Jiawei Mao, Jeremy Hsu, Jinman Kim

**Affiliations:** 1 Biomedical Data Analysis and Visualisation (BDAV) Lab, School of Computer Science, The University of Sydney, Camperdown, NSW 2050 Australia; 2 Trauma Service, Westmead Hospital, Westmead, NSW 2145 Australia; 3 Department of Surgery, Faculty of Medicine and Health, The University of Sydney, Camperdown, NSW 2050 Australia

**Keywords:** Fracture localization, Mixed reality, Surgical stabilization of rib fractures, Visualization system, Patient-specific holograms, HoloLens

## Abstract

In surgical stabilization of rib fractures (SSRF), the current standard relies on preoperative CT imaging and often incorporates ultrasound (US) imaging. As an alternative, mixed reality (MR) technology holds promise for improving rib fracture localization. This study presents an MR-based visualization system designed for SSRF in a clinical setting. We developed RibMR – a visualization system using an MR head-mounted display that projects a patient-specific 3D hologram onto the patient. RibMR enables the localization of rib fractures in relation to the patient’s anatomy. We conducted phantom study using a human mannequin, a preclinical study with two healthy patients, and clinical study with two patients to evaluate RibMR and compared it to US practice. RibMR localized rib fractures with an average accuracy of 0.38 ± 0.21 cm in phantom, 3.75 ± 2.45 cm in preclinical, and 1.47 ± 1.33 cm in clinical studies. RibMR took an average time (minutes) of 4.42 ± 0.98 for the phantom, 8.03 ± 3.67 for the preclinical, and 8.76 ± 0.65 for the clinical studies. Compared to US, RibMR located more fractures, including fractures occluded by other structures, with higher accuracy, faster speed, and improved localization rate. All participating surgeons provided positive feedback regarding accuracy, visualization quality, and usability. RibMR enabled accurate and time-efficient localization of rib fractures and showed better performance compared to US. RibMR is a promising alternative to US for localizing rib fractures in SSRF.

**Supplementary Information:**

The online version contains supplementary material available at 10.1007/s10278-024-01332-2

## Introduction

Rib fractures are associated with approximately 10% of trauma injuries and a mortality rate as high as 20% [1]. The mainstay of treatment focuses on pain relief and respiratory support to prevent the development of pneumonia. Surgical stabilization of rib fractures (SSRF), involving the use of titanium plates, is a major intervention in selected cases that improves outcomes [1]. This procedure involves an incision designed based on fracture characteristics and locations identified from preoperative computed tomography (CT) images [2]. However, the limitation of SSRF is the need for a large skin incision and tissue dissection to access the rib fracture, which is extended and can complicate postoperative recovery if the fractures are not precisely localized [2, 3].

Relying solely on CT images to localize rib fractures has the limitation that the surgeon needs to mentally map the fractures seen on the screen onto the patient’s body [2]. This is a difficult process because patient positioning in the operating room (OR) differs from that in the CT images [3]. Therefore, complementary methods such as ultrasound (US) and video-assisted thoracoscopy (VATS) are used in addition to CT images to improve rib fracture localization [2]. US is used after anesthesia is induced and the patient is positioned in the OR. A surgeon scans each rib along its longitudinal axis to detect cortical disruption, which is indicative of fractures [2]. Martin et al. [3] found that US reduced the incision length and operative time, and improved postoperative pain management. However, the accuracy of US can vary depending on the location of the fracture and the patient’s body morphology [4, 5]. For example, US has limited access to the subscapular and infraclavicular areas [5], and its limited soft tissue penetration limits its use in obese patients or those with large breasts [6]. In addition, the use of US is time-consuming and requires an experienced surgeon to interpret the images [4, 5]. VATS is used after induction of anesthesia and intubation for single lung ventilation, involving thoracic trocar placement. It allows the refinement of fracture locations based on their direct visualization in the thoracic cavity [7]. Schots et al. [7] noted advantages of VATS, including reduced muscle destruction and shorter postoperative recovery time. However, VATS requires the patient to be eligible for single-lung ventilation [7]. It is a time-consuming and costly method that requires skilled surgeons experienced in thoracic surgery [7].

Mixed reality (MR) technology can provide an alternative for rib fracture localization by integrating virtual elements into a physical environment as a hologram [8]. MR can be used to overlay the patient’s CT-derived three-dimensional (3D) rib model onto the patient’s body. This eliminates the need for mental mapping between the CT images and the patient. It also provides a wider field of view (FoV) compared to US, allowing for more efficient fracture localization. MR can enable accurate localization of rib fracture without the need for intubation, single-lung ventilation, or thoracic trocar insertion which are required for VATS.

Recent advances in MR head-mounted display (HMD) technology have fostered the development of MR-based systems for surgical applications [8]. In these systems, a patient model derived from medical images is superimposed on the patient’s body as a hologram. Depending on the application, manual, semi- or automatic alignment of the patient model onto the patient (model-patient alignment) is used [8]. Teatini et al. [9] demonstrated an MR system for orthopedic surgery where a CT-derived lower limb model was semi-automatically aligned to the phantom using markers and an additional tracking device. van Doormaal et al. [10] proposed an MR system for neurosurgery where a magnetic resonance imaging (MRI)-derived head model was semi-automatically aligned to the patient using fiducial markers. Gibby et al. [11] guided percutaneous screw placement using a commercial MR system that automatically aligns lumbar models using 3D features of the model and phantom surfaces. For a sarcoma resection procedure, Moreta-Martinez et al. [12] proposed an MR system that uses a 3D-printed fiducial marker clamped to the patient’s tibia for semi-automatic alignment. Gsaxner et al. [13] presented a head and neck surgery system that automatically aligns a patient model using 3D facial feature points on the model and patient. Scherl et al. [14] manually aligned patient model to patients for tumor resection.

Despite the potential of MR for surgical applications, we have identified five distinct requirements for SSRF that render existing systems unsuitable. First, preoperative CT scans for SSRF are typically acquired in an emergency, precluding modifications to the scanning protocol. Consequently, methods that depend on pre-scan attachment of registration markers and their maintenance until surgery (e.g., van Doormaal et al. [10]) are infeasible in this context. Second, SSRF aims to minimize incisions to enhance patient outcomes, such as reducing postoperative recovery time [15]. Therefore, methods requiring exposure of rigid anatomical structures necessitate additional incisions and may be impractical for SSRF (e.g., Moreta-Martinez et al. [12]). Third, SSRF is an emergent trauma surgery typically scheduled within one or two days to resuscitate and stabilize the patient, with patients assigned to any available operating room. Thus, systems requiring complex setups and cumbersome calibration, such as additional tracking devices (e.g., Teatini et al. [9]), are unlikely to be suitable. Fourth, the surgical area in SSRF—the patient’s torso—lacks distinctive surface features for establishing correspondence between the patient model and the patient. Consequently, methods that rely on unique anatomical features for alignment (e.g., facial features in Gsaxner et al. [13]) may not be applicable. Finally, the patient’s torso undergoes significant deformation based on surgical positioning, violating the assumption that the torso in the patient model matches the intraoperative anatomy. This impedes the use of MR systems that rely on rigid alignment (e.g., Gibby et al. [11]). Consequently, no existing MR system has been specifically designed to address all five of these requirements, thereby limiting their effectiveness in supporting surgeons with the intraoperative localization of rib fractures in SSRF.

**Fig. 1:**
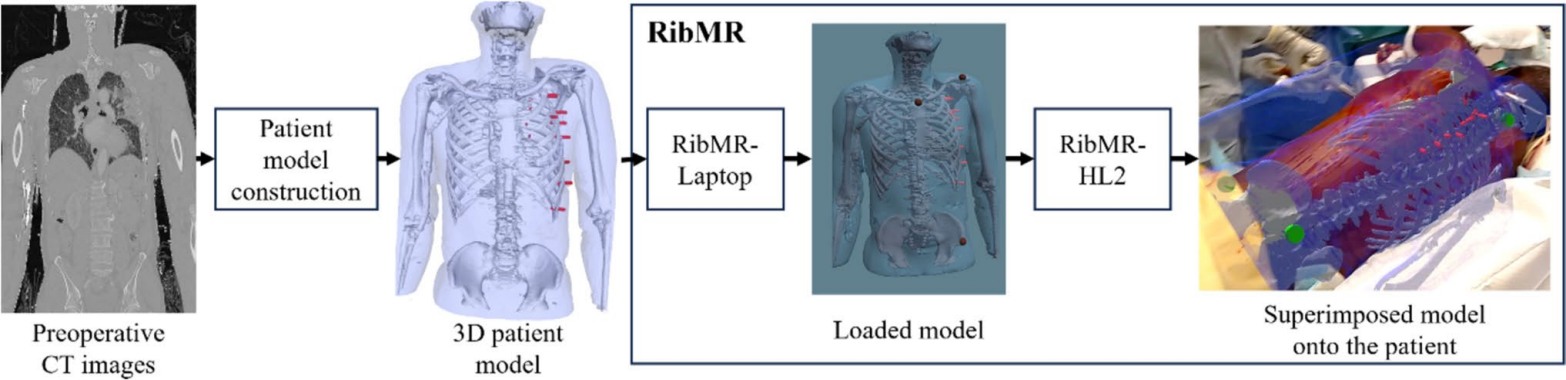
An overview of patient model construction and RibMR with standard preoperative CT images

To address these specific requirements, we introduce RibMR, a novel MR-based visualization system, specifically designed for intraoperative rib fracture localization in the SSRF. As illustrated in Fig. 1, RibMR employs patient model derived from standard emergency CT images and aligns it to the patient in a non-invasive, intuitive, and time-efficient manner. RibMR uniquely satisfies the critical requirements of SSRF. These tailored solutions ensure RibMR’s compatibility with the urgent and variable conditions of SSRF and its seamless integration into the clinical workflow. Furthermore, RibMR has been rigorously evaluated through phantom study as well as preclinical and clinical studies, demonstrating its effectiveness and reliability in real-world surgical environments. The key contributions of this study are:

- Introduction of a new semi-automatic model-patient alignment procedure that accounts for the patient’s torso deformation by using five bony landmarks on the patient and the singular value decomposition (SVD) algorithm [16], without requiring modifications to scanning protocols, thereby ensuring compatibility with emergency settings.
- Development of a semi-automatic segmentation and model construction pipeline for RibMR.
- Iterative design and implementation of RibMR within an SSRF workflow in a Level 1 trauma center.

Evaluation of our prototype RibMR system included three experiments: a phantom study, and preclinical and clinical studies in humans. The system’s accuracy and speed of rib fracture localization were evaluated and compared to current US practice. Feedback on RibMR’s model-patient alignment, patient model visualization, and usability was obtained from three surgeons.

## Materials and Methods

###  Preprocessing: Segmentation and 3D Patient Model Construction

This section describes the preprocessing steps to prepare CT images for use in RibMR. The preprocessing consists of two steps: (1) segmenting the body, bones, and rib fractures and (2) reconstructing the segmentations into a 3D patient model. While the following subsections detail the techniques we employed, alternative methods yielding equivalent results are also acceptable.

#### Segmentation of Body, Bones, and Rib Fractures

We collected CT images obtained using the standard scanning protocol of the Level 1 trauma center and its referring medical institutions. The standard scanning protocol produced multiple sets of CT images (CT series) representing different anatomical sections (e.g., chest and abdomen).

**Fig. 2:**
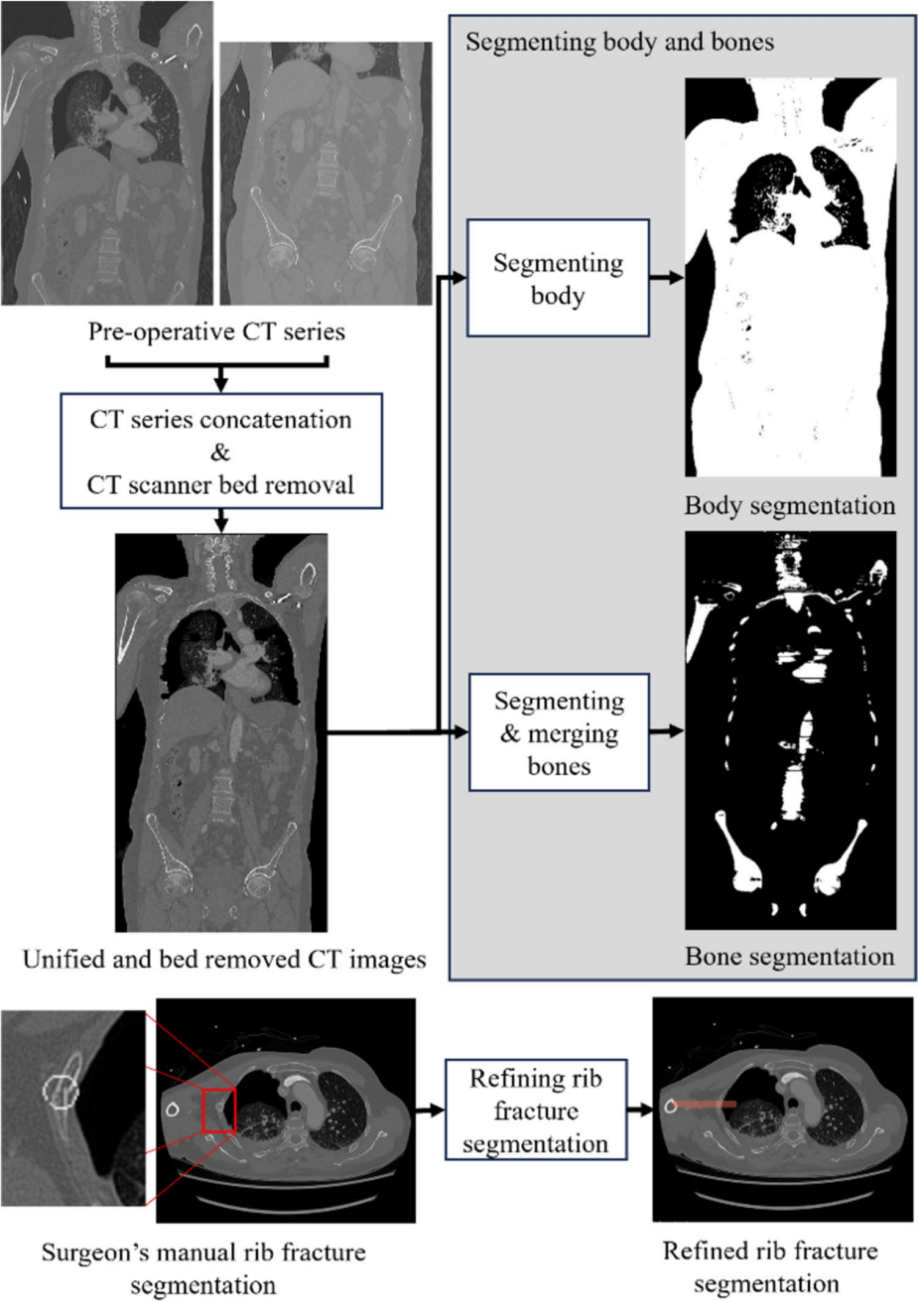
Overview of the patient body, bone, and rib fracture segmentations

The CT series were concatenated to form a unified set, incorporating as many stable bony landmarks as possible, as illustrated in Fig. 2. The CT scanner bed was removed from this unified set using a 2D U-Net model [17] trained on our dataset specifically for bed segmentation [18]. The bed-removed CT images were segmented using a thresholding technique with an empirically derived value set at the 20th percentile of the minimum-maximum intensity value range. This thresholding created a body segmentation mask from the remaining image. Bone segmentation was performed automatically using two 2D U-Net models [17]: (i) for rib cage segmentation using the Usevilla Bone and Muscle dataset [19] and (ii) segmentation focusing on the overall bone structures using CT-ORG dataset [20]. The resulting segmentations were combined using the logical OR operator. All rib fractures were manually segmented by a surgeon and refined by a system development team to ensure alignment with other segmentations and to extend the rib fracture indicator perpendicularly to the skin. The performance of bone and body segmentations is detailed in the Appendix (Supplementary Material 1).

#### 3D Patient Model Construction

**Fig. 3:**
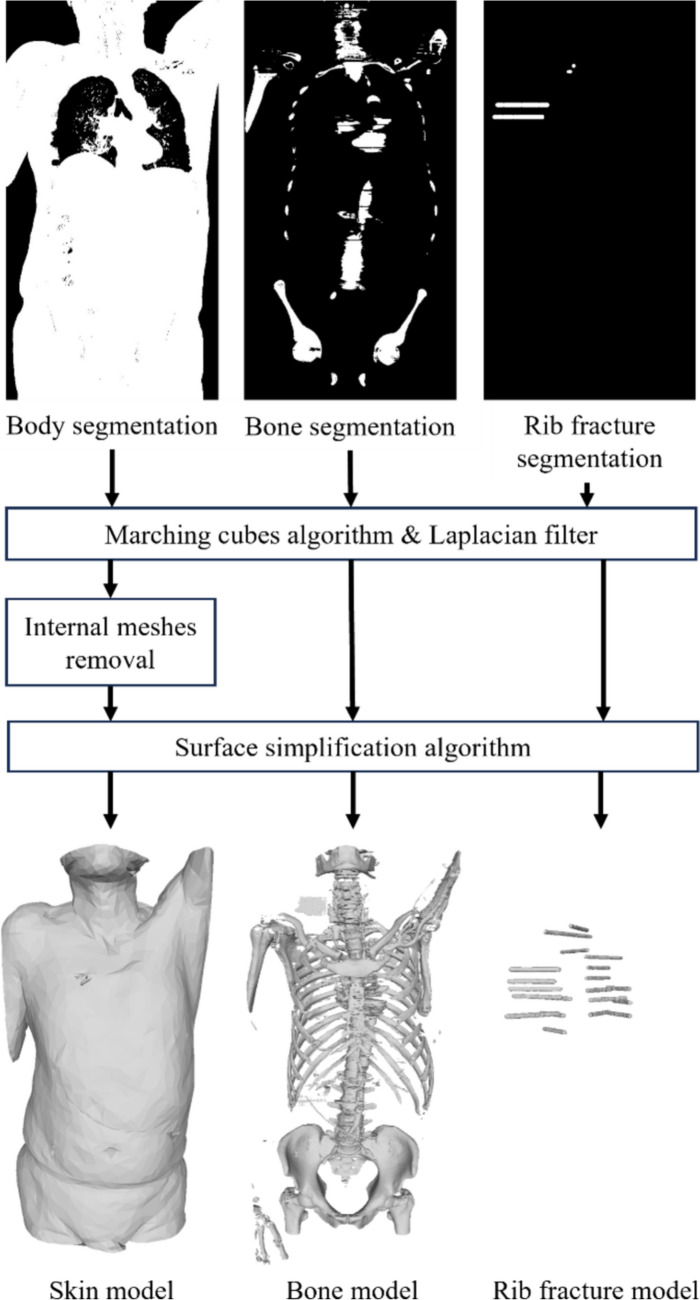
Overview of constructing the 3D patient model – the skin, bone, and rib fracture models

The segmentations were transformed into a 3D patient model (i.e., skin, bone, rib fracture models) using the Marching Cubes algorithm [21] and the Laplacian filter (Fig. 3). Within the skin model, unnecessary internal faces (e.g., respiratory system) were removed by discarding faces that were always in shadow using the Ambient Occlusion technique [22]. The surface simplification algorithm [23] was used to reduce the faces of the models for computational efficiency. The patient model was reviewed and manually corrected by a system development team with a surgeon, including model refinement and artifact removal.

### RibMR

**Fig. 4:**
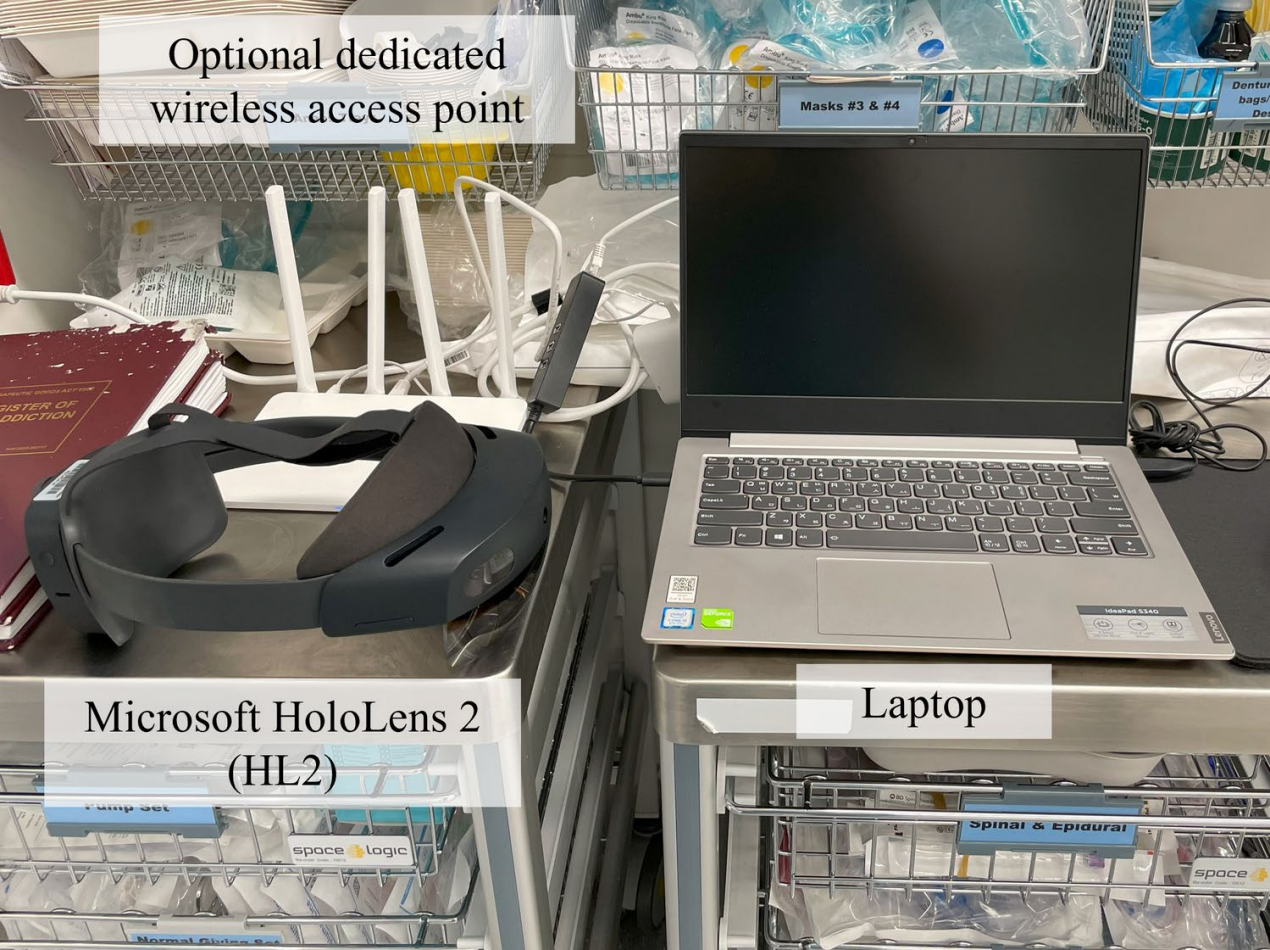
Hardware configuration of RibMR set on the medical cart in the OR

RibMR has been iteratively designed and developed with surgeons from the Level 1 trauma center to comply with requirements of SSRF and surgeons in the session. The hardware setup for RibMR consists of a laptop, Microsoft HoloLens 2 (HL2), and an optional dedicated wireless access point, as illustrated in Fig. 4. This hardware can be placed on a cart for portability and easily set up in any OR.

**Fig. 5:**
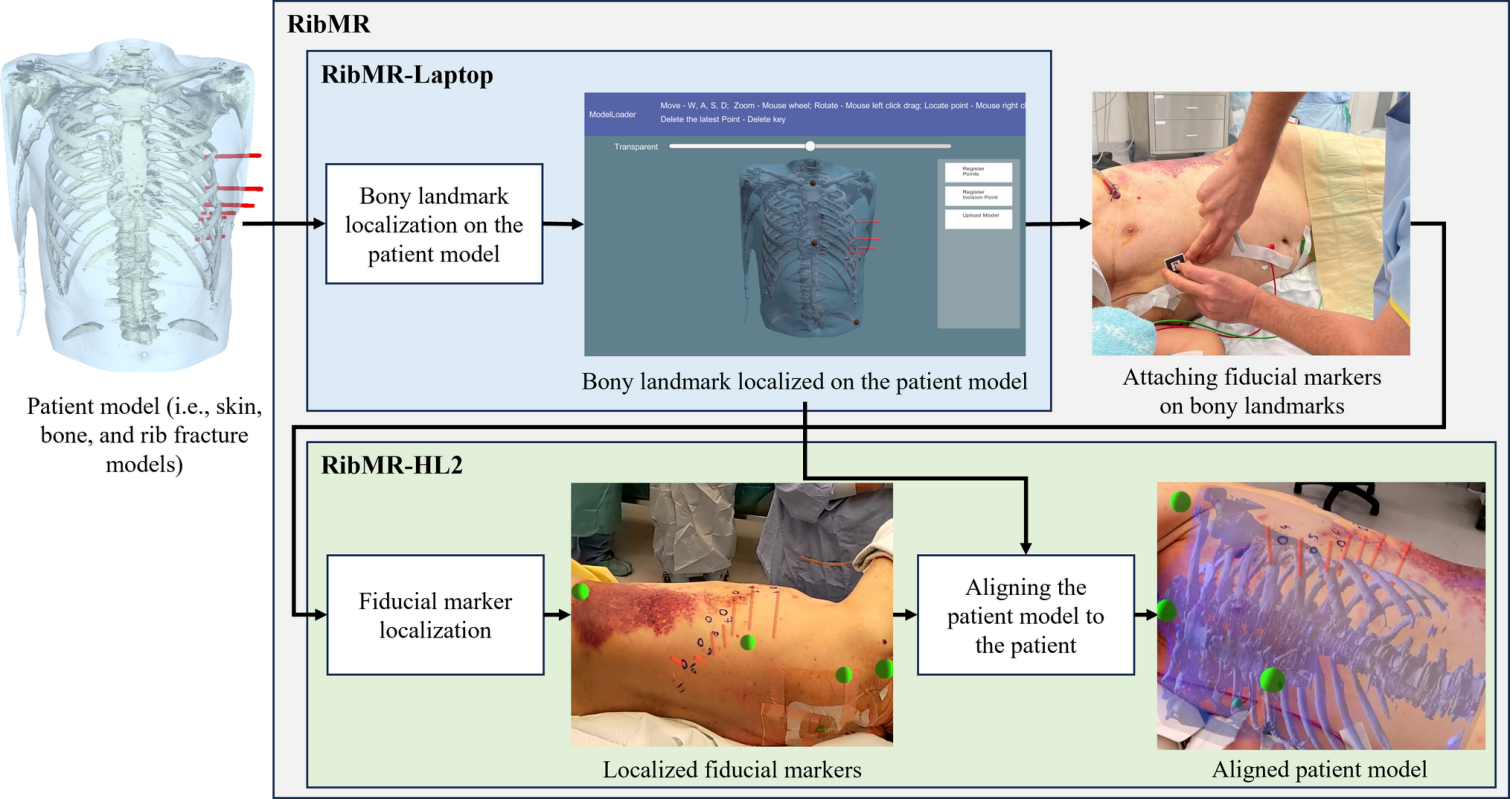
Overview of the RibMR and its semi-automatic model-patient alignment procedure

RibMR consists of two software applications: RibMR-Laptop and RibMR-HL2 (Fig. 5). The RibMR-Laptop contains a user interface (UI) for loading the patient model. RibMR-HL2 receives the patient model from the RibMR-Laptop, renders it into a hologram, and aligns it to the patient.

RibMR has a semi-automatic model-patient alignment procedure that uses five bony landmarks. This procedure consists of 3 processes (Fig. 5) which are: (1) localizing the bony landmarks on the patient model; (2) attaching and localizing the fiducial markers; and (3) aligning the patient model to the patient. This alignment procedure is intended to be performed during the SSRF. RibMR-Laptop and RibMR-HL2 were used accordingly throughout the process.

#### Bony Landmark Localization on the Patient Model Process

**Fig. 6:**
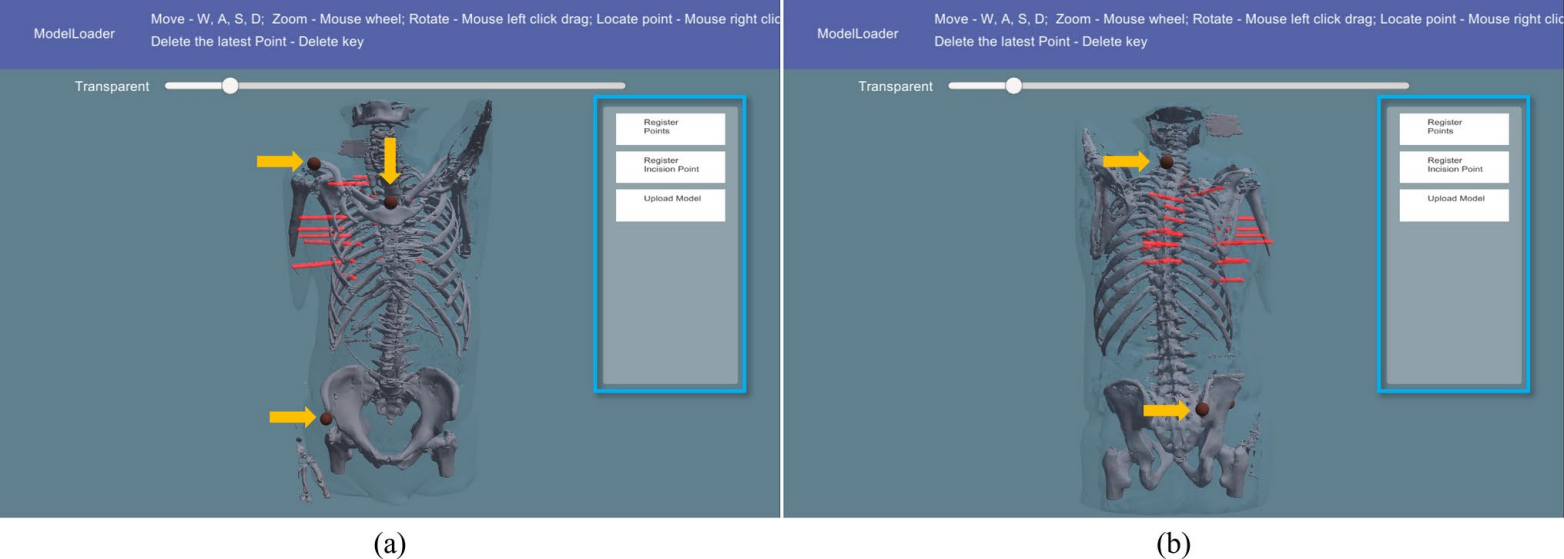
RibMR-Laptop with the patient model loaded and viewed from front (**a**) and back (**b**). The transparency of skin model has been adjusted to visualize the bone model distinctively

The patient model was loaded onto the RibMR-Laptop. The skin, bone, and rib fracture annotation models were visualized in semitransparent blue, opaque white, and semitransparent red, respectively (Figs. 5 and 6). The RibMR-Laptop has a control UI (as illustrated in Fig. 6 blue boxes) to load the patient model, localize the bony landmarks, and upload the patient model and location of bony landmarks to its internal server.

After evaluating the loaded patient model and condition of the patient in the OR, five bony landmarks were selected from a predefined list of 10 candidate bony landmarks. These candidates were selected based on their rigidity during patient position changes and their distinctiveness within the patient model and the patient’s body. The selected candidates include the suprasternal notch, left and right acromioclavicular (AC) joints, xiphisternum (Xiphi), the left and right anterior superior iliac spine (ASIS), C7 and T1 vertebrae, and left and right posterior superior iliac spine (PSIS).

The user localized five bony landmarks on the patient’s skin model (referred to as the L_Model_) by clicking on the designated positions, while the bone and rib fracture annotation models were visualized through the skin model, as illustrated in Fig. 6. The patient model could be freely translated, rotated, and scaled as a set, with adjustable transparency of the skin model. Each landmark localization was confirmed by a 5 mm diameter red sphere (yellow arrows in Fig. 6) at the clicked location, adjusted if necessary. After the confirmation, the patient model and the location of the body landmarks were packaged as a data stream and uploaded to the RibMR-Laptop’s internal server to be transferred to the RibMR-HL2.

#### Fiducial Marker Attachment and Localization Process

After positioning the patient in the OR, five rectangular fiducial markers (3.2 × 3.2 cm) were placed on the patient, referred to as L_Patient_; these L_Patient_ corresponded to L_Model_. The markers were labeled as A1, B2, C3, D4, and E5 using patterns from ARToolKit [24] following the sequences in L_Model_.

**Fig. 7:**
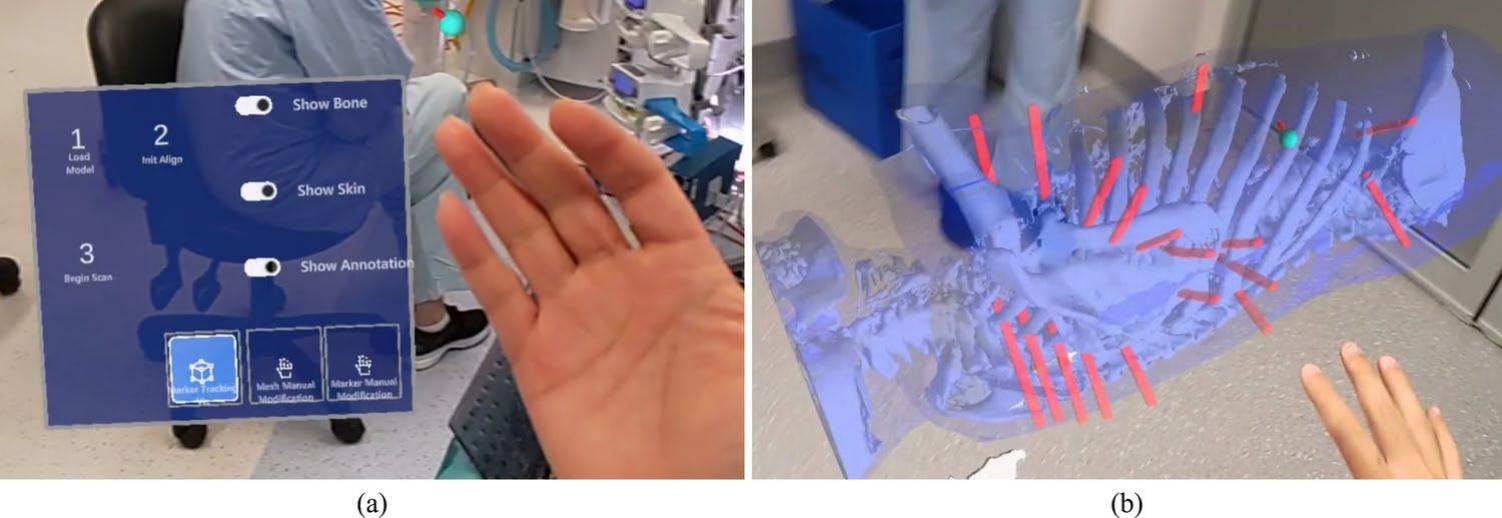
The control UI of RibMR-HL2 (**a**) and the loaded patient model on RibMR-HL2 before the alignment (**b**)

The user wore the HL2 and started the RibMR-HL2. The RibMR-HL2 has a control UI that appears next to the user’s palm, as illustrated in Fig. 7(a). The control UI provides buttons to load the patient model from the RibMR-Laptop, align the loaded patient model to the patient, visualize the patient surfaces captured by HL2, change the visibility of skin, bone, and rib fracture models and a marker indicator, and toggle manual adjustment of the 3D patient model and the marker indicator. By pressing the patient model loading button (“1 Load model” button in Fig. 7), the patient model and the L_Model_ were transferred to the RibMR-HL2 from the RibMR-Laptop’s internal server. The patient model appeared as a hologram in the OR, as illustrated in Fig. 7(b).

The user attempted to locate all five fiducial markers on the RibMR-HL2. The RibMR-HL2 employed ARToolKit [24] to detect and localize these markers within the HL2’s camera video stream. ARToolKit determined the markers’ positions and orientations by analyzing perspective distortion, extracting corner points, and calculating the homography matrix using the actual marker size. With camera parameters such as focal length and optical center, ARToolKit accurately estimated the markers’ poses and scales, reporting the results back to RibMR-HL2.

Upon detecting and localizing a marker, RibMR-HL2 displayed a 20 mm diameter green holographic sphere (Fig. 5) at the center of each marker to confirm its location. If a sphere was off-center, the user repositioned it by moving closer or improving its visibility with a flashlight. Once a sphere was correctly positioned, the corresponding marker was removed. After all markers were removed, the user checked the overall placement of the spheres. If misplacement was observed, the corresponding marker was reattached and relocalized, or the localized location was manually corrected. These locations became L_Patient_.

#### Aligning the Patient Model to the Patient Process

The alignment between the loaded patient model and the patient was initiated by pressing the alignment initiation button (“2 Init Align” button in Fig. 7). RibMR-HL2 estimated a rigid transformation to align the loaded patient model to the patient using the landmarks L_Model_ and L_Patient_, based on the SVD algorithm [16]. The alignment process is mathematically grounded in the least-squares fitting problem for two sets of 3D points under point correspondences, as described by Arun et al. [25]. The objective of this fitting problem is to find a transformation (rotation *R* and a translation *T*) that minimizes the sum of squared differences between L_Model_ and L_Patient_, expressed as:

1 $$Transformation=\underset{Transformation}{arg\;min}\sum_{i=1}^5\left\|L_{Patient,i}-\left(R\cdot L_{Model,i}+T\right)\right\|_2^2$$

To solve this problem, the centroids of both point sets L_Model_ and L_Patient_ were calculated. These centroids served as reference points for centering L_Model_ and L_Patient_. The covariance matrix *H* was then computed from the centered L_Model_ and L_Patient_. The SVD algorithm applied on *H* produced two unitary matrices *U* and *V*^*T*^, which are used to compute the optimal *R*. Optimal *T* was derived by aligning the centroids after applying *R*.

The steps for this alignment process are outlined in Algorithm 1:

**Algorithm 1:**
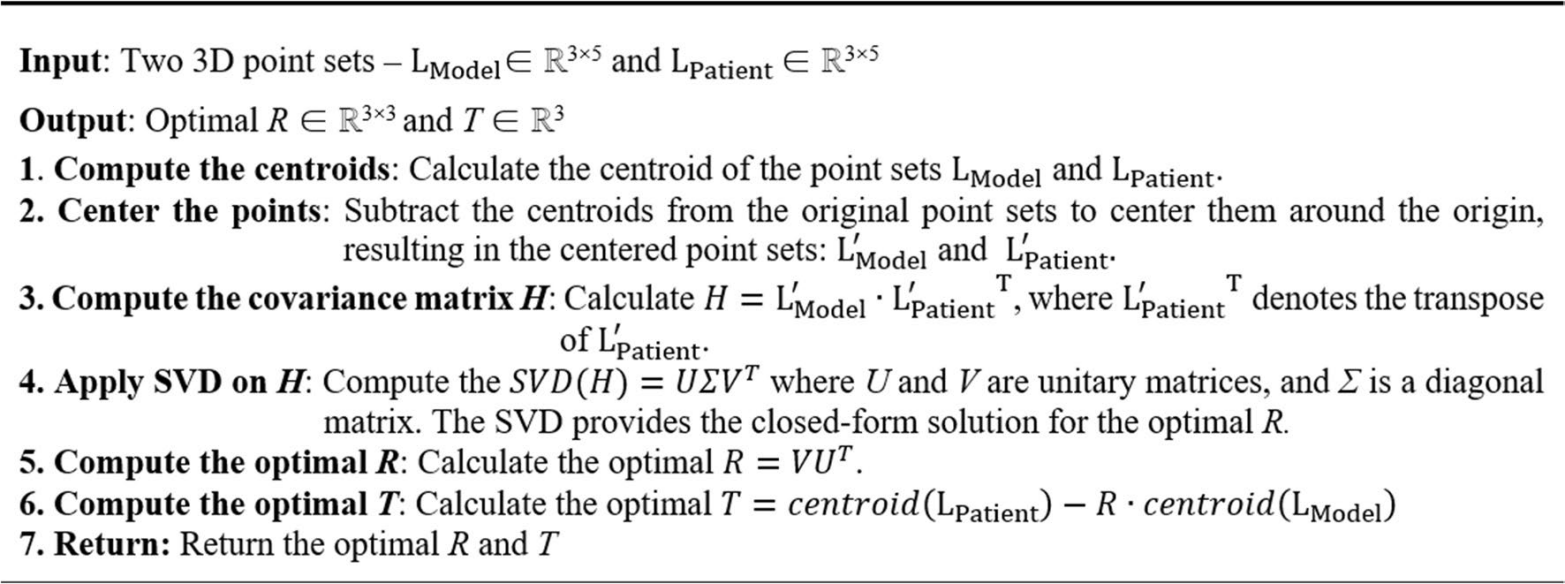
SVD-based least squares solution for optimal *R* and T

Algorithm 1 was executed exclusively within RibMR-HL2, and the resulting optimal *R* and *T* were applied to align the patient mode with the patient. This automated alignment process ensures accurate model-patient alignment. The model-patient alignment procedure was concluded once the optimal 𝑅 and 𝑇 were applied.

### Implementation

The preprocessing steps, “Segmentation and 3D Patient Model Construction”, were implemented using U-Net implementation [26], PyTorch library [27], 3D Slicer [28], and MeshLab [29], on a PC with Intel i7-8700K CPU, 64GB memory, and NVIDIA 2080Ti GPU.

The RibMR system was developed using C# and the Unity Engine (version 2019.4.28f1) [30]. RibMR-Laptop employed the Runtime OBJ Importer [31] in loading the patient model file to the RibMR-Laptop and was deployed on a laptop with an Intel i5-8265U CPU, 20GB memory, and NVIDIA MX230 GPU. RibMR-HL2 employed the Microsoft Mixed Reality Toolkit (version 2.4.0) [32] for the control UI, the HoloLensARToolKit Library (version 0.3) [33] for the marker detection and localization, and the MathNet Library [34] for the calculations in Algorithm 1.

## Experimental Settings

RibMR underwent the phantom study using a human mannequin, the preclinical study with two healthy patients, and the clinical study with two patients to evaluate its accuracy, localization rate, and speed in localizing rib fractures. The accuracy was assessed by measuring the distance between the actual rib fractures and those localized by RibMR or US, as commonly employed in various studies [35]. The localization rate denotes the percentage of rib fractures identified by RibMR or US, while speed was evaluated by measuring the time required for rib fracture localization. In the preclinical and clinical studies, we collected verbal feedback from participating surgeons on model-patient alignment, model visualization, and system usability.

The phantom study provided a controlled environment in which body deformations were removed and fixed L_Model_, L_Patient_, and rib fracture locations were used. This controlled environment validated RibMR’s model-patient alignment procedure in practice before the preclinical and clinical studies with human patients.

The preclinical study on two volunteer patients without rib fractures was designed to evaluate RibMR in humans and test its suitability for human use in a controlled simulated OR environment. Patients in this environment were in surgical positions with better body visibility compared to the OR and were therefore not affected by occlusions from medical equipment and/or personnel. Additionally, this environment allows evaluation to be free of the mental and physical stressors inherent in the surgical environment.

The clinical study of two patients was conducted to evaluate RibMR in the context of SSRF and a clinical setting within the existing surgical workflow.

For the studies, approval was granted by the Ethics Committee of the Western Sydney Local Health District (2021/ETH00209). Informed consent was obtained from all participating patients.

### Phantom Study

A human torso mannequin (39.0 × 22.4 × 67.0 cm) was used as the patient phantom. 32 metal washers (a circular piece of metal) of 7 mm diameter were attached to the phantom (see Appendix for figure and locations) to use the fixed L_Model_, L_Patient_, and rib fracture locations and to measure the surface alignment accuracy in the study. The phantom models were created from CT images of the phantom with the metal washers by manually segmenting the surface and the washers using thresholding due to their different composition from the human.

A computer science student with experience in HL2 and RibMR participated in the phantom study. The phantom was positioned in the supine, left lateral decubitus, and right lateral decubitus positions. Five simulated L_Model_/L_Patient_ pairs and 16 selected simulated rib fractures (see Appendix for locations) were used to align the phantom model and evaluate accuracy at each phantom position, respectively. RibMR rib fracture localization accuracy was determined by measuring the shortest surface distances between RibMR-located fractures and their washer-marked positions (see Appendix for figure). Rib fracture localization time was recorded from loading of the phantom models to completion of rib fracture localization.

### Preclinical Study

Three surgeons, including one senior surgeon experienced in SSRF (referred to as S1) and two surgical trainees (referred to as S2 and S3) participated in the preclinical study. S2 and S3 also participated as two healthy patients without rib fractures (referred to as P2 and P3 – see Appendix for demographics). S1 and S2 had previous experience with HL2 while S3 reported no experience with HL2 or any other HMD.

Before the study, the surgeons received a hands-on introduction to RibMR from the system development team, and HL2 was calibrated to their eyes. Additionally, the segmentation results were visually inspected on the 3D patient model by the surgeons using our RibMR. In the study, S1 and S2 assumed the role of a surgeon while P3 was positioned supine on a simulated operating table (see Appendix for figure). For P2, S1 and S3 assumed the role of surgeon while P2 was positioned right lateral decubitus. CT images and patient skin and bone models were obtained before the study.

 As there were no rib fractures in P2 and P3, six bony landmarks (see Appendix for locations) were used as simulated rib fractures. The location of the six bone landmarks was marked on the patient by palpation using an invisible marker. Surgeons were asked to perform the model-patient alignment procedure and mark the simulated rib fractures on the patient using RibMR. After marking, verbal feedback was collected from the surgeons.

We evaluated the accuracy of RibMR by measuring the shortest surface distances between RibMR-localized and palpation-localized simulated rib fractures for each surgeon. We also recorded the time spent using RibMR from loading the patient model to completion of fracture marking.

### Clinical Study

Two surgeons participated in the clinical study: S1 and S2 from the preclinical study, S3 was unavailable. In this study, S2 only used RibMR to locate the rib fractures, while S1 only used US according to the standard SSRF protocol. S2 had experience with RibMR from the preclinical study. S1 had previous experience with US in SSRF.

Two patients were recruited between September 2022 and April 2023 (see Appendix for demographics). All patients undergoing SSRF with at least three of the candidate body landmarks available were eligible for the study. Participating patients were in different degrees of lateral decubitus position in the OR. Patient CT images and models were obtained one to two days before SSRF. The surgeon using RibMR (RibMR surgeon) received a hands-on introduction to RibMR from the system development team before the first SSRF of the study. Prior to each SSRF, the participating surgeon visually inspected the segmentation results on the 3D patient model using the RibMR.

In each SSRF, we asked two surgeons to use RibMR and US to locate rib fractures (see Appendix for figure). HL2 was calibrated to the eyes of the RibMR surgeon. The RibMR surgeon determined L_Model_ and L_Patient_ (see Appendix for locations) based on their accessibility and visibility in the CT images and the patient, and aligned the patient model to the patient through the model-patient alignment procedure. Rib fractures were marked independently on the patient using RibMR and US. The surgeon using US marked the rib fractures according to their standard SSRF workflow. Verbal feedback was obtained from the RibMR surgeon. The remaining SSRF followed the standard protocol regardless of RibMR marking.

The shortest distance from the rib fracture to the RibMR and US markers was measured intraoperatively using a surgical ruler before suturing. Therefore, the shortest distance was only measured for rib fractures that were fixed during SSRF. The comparison of accuracy between RibMR and US was performed only for rib fractures marked by both methods and fixed during SSRF. For RibMR, the time from loading the patient model to the end of fracture marking was recorded, whereas for US, the time from first probe contact to the end of fracture marking was recorded. The number of fractures identified by RibMR and US was also recorded.

### Statistical Analysis

Descriptive and analytical statistical methods were used for all studies, including mean ± standard deviation (SD). The statistical significance was determined by one-way analysis of variance (ANOVA) followed by Tukey’s honestly significant difference (HSD) test and two-sample t-test [36], with a significance level of *p* < 0.05. All statistical calculations were performed with Microsoft Excel.

## Results

### Rib Fracture Localization Accuracy

#### Phantom Study

RibMR achieved an average rib fracture localization accuracy of 0.38 ± 0.21 cm in the phantom study. All 48 simulated rib fractures (16 simulated rib fractures × 3 phantom positions) were located (100% localization rate), and their shortest surface distances were measured. The accuracy at each phantom position can be found in Appendix.

#### Preclinical Study

An average rib fracture localization accuracy of 3.75 ± 2.45 cm was observed in the preclinical study. Surgeons localized 22 out of 24 simulated rib fractures (91.67% localization rate) across 2 patients, with each having 2 surgeons and 6 simulated rib fractures. S3 could not mark two fractures (left AC joint and left scapular tip) on P2 due to the visualization issue in RibMR, where these fractures appeared outside the patient’s body. The accuracy of each patient position and surgeon can be found in Appendix.

#### Clinical Study

In the clinical study, RibMR localized 22 out of 25 rib fractures (88% localization rate) across the two patients, with one patient having 14 fractures and the other 11. US localized 14 rib fractures (56% localization rate). RibMR successfully marked all fractures identified by US. A two-way t-test could not find statistical significance (*p* > 0.05) in the number of rib fractures localized by RibMR versus US.

**Fig. 8:**
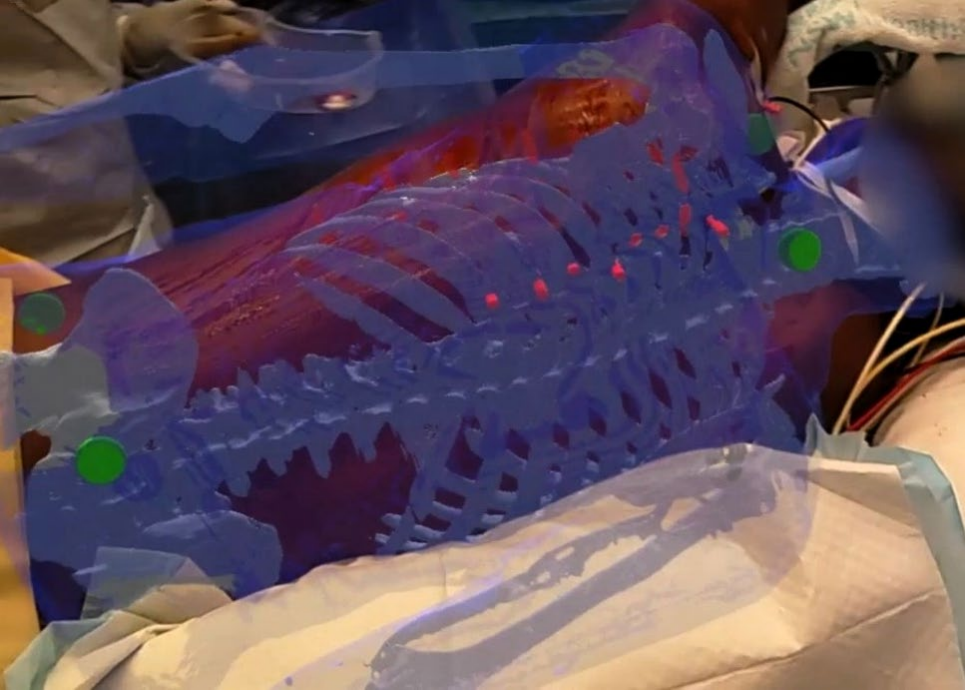
Visualization of a patient model in RibMR after the model-patient alignment

Figure 8 shows the visualization of the patient model in RibMR after the model-patient alignment procedure. An example video of two visualizations can be found in the Supplementary Material 2.

Of the 22 RibMR and 14 US rib fracture markers, the shortest distances were measured for 17 RibMR and 13 US markers. Other markers could not be measured because the corresponding fractures were not fixed during SSRF. Of the rib fractures measured, all rib fractures marked by US were localized by RibMR, while four rib fractures marked by RibMR were not localized by US.

RibMR had an average accuracy of 1.47 ± 1.33 cm in the clinical study. Additionally, for the four rib fractures missed by US, RibMR showed an average accuracy of 1.75 ± 2.36 cm.

For the comparison between RibMR and US, the shortest distances of 13 rib fractures marked by both methods were used. The average accuracy of RibMR was 1.38 ± 0.96 cm, while the accuracy of US was marginally worse (two-sample t-test *p* > 0.05) at 3.46 ± 5.46 cm. The average accuracy of RibMR and US in each SSRF can be found in Appendix.

### Rib Fracture Localization Times

RibMR took an average of 4.42 ± 0.98 min in the phantom study, 8.03 ± 3.67 min in the preclinical study, and 8.76 ± 0.65 min in the clinical study. In the clinical study, US resulted in an average of 9.99 ± 6.39 min which was marginally slower than RibMR (two-sample t-test *p* > 0.05). In terms of time spent per fracture, RibMR required an average of 0.85 ± 0.27 min in the clinical study, while US required marginally more time (two-sample t-test *p* > 0.05) with an average of 1.49 ± 0.17 min. The time spent by each phantom position in the phantom study, by each surgeon in the preclinical study, and by each SSRF in the clinical study can be found in Appendix.

### Verbal Feedback

#### Model-patient Alignment

In the preclinical study, the surgeons observed an approximate offset of 1–2 cm during visual inspection of the aligned patient model. S1 and S2 commented that this is an acceptable level of accuracy for designing an incision in the SSRF and confirmed this by palpation. However, during marking, S3 expressed that the left AC joint and left scapular tip representatives within the aligned bone model were outside of P2.

In the clinical study, S2 initially observed no discernible misalignment and confirmed that the ribs were palpated based on their appearance in RibMR. In the first SSRF, an offset of approximately 1–2 cm to the front of the patient and 1 cm to the side was described. In the second SSRF, a slight deviation of the patient’s spine of 2 cm to the floor was observed, along with a slight tilt to the patient’s left side. However, it was commented that the quality of alignment in the second SSRF was high, especially around the patient’s rib cage.

#### Model Visualization in RibMR

In the preclinical study, S1 and S2 reported an impairment of depth perception that made it difficult to accurately estimate the distance between the rib and the skin. S2 perceived P3’s ribs to be visualized deeper than they actually were. S1 attributed this impairment to difficulty seeing the patient’s skin due to the aligned patient model. S3 was able to count the ribs on the aligned bone model but noted a shorter sternum than in reality.

In the clinical study, the bone models were commented on as providing good contrast and clear visualization of the ribs and allowing identification and localization of fractures. However, in the second SSRF, the bone models were found to be missing part of non-fractured ribs due to segmentation errors.

Overall, the rib fracture annotation model was appreciated for its effectiveness in identifying and locating fractures in the patient’s body. Clear visibility and the ability to objectively mark fractures were noted as beneficial features.

#### System Usability

All surgeons expressed satisfaction with the usability, workflow, and UI of the system, describing it as easy to use, straightforward, and effective. S2 noted a learning curve in using the system, particularly in placing and localizing fiducial markers, suggesting proficiency over time. A primary recommendation was to reduce the number of fiducial markers to improve usability.

## Discussion

SSRF is the primary approach for treating rib fractures, but it involves extensive skin incisions and tissue dissection when precise rib fracture localization is not available. We demonstrated that RibMR enabled surgeons to identify and localize rib fractures in four patients in both simulated and real OR settings, and resulted in higher accuracy, higher localization rate, and faster speed than US in SSRF.

We made the following key findings: (1) RibMR showed an average rib fracture localization accuracy within sub-centimeters in the phantom study and within 3 and 1 cm in the preclinical and clinical studies. These offsets were considered useful for incision design; (2) RibMR identified a greater number of rib fractures, achieved a higher localization rate, and showed better accuracy than US; (3) RibMR's rib fracture localization was completed faster than US; (4) Impairment of depth perception and occasional loss of partial rib segments were observed in the RibMR's patient model visualization. Despite this, the surgeons successfully located the rib fractures and, (5) RibMR received positive feedback on its ease of use and the potential to improve the effectiveness of the system with additional training.

RibMR demonstrated sub-centimeter accuracy (0.38 ± 0.21 cm) in the phantom study. However, in human patients, the system showed accuracy deficits of 3.37 cm in the preclinical study and 1.09 cm in the clinical study. We attribute the offset between the preclinical and clinical studies to S3 in the preclinical study which had a significantly lower accuracy (one-way ANOVA and Tukey HSD tests *p* < 0.05) of 7.25 ± 3.37 cm than S1 and S2. However, the decrease in accuracy with human patients compared to the phantom is attributed to patient body deformations, hardware limitations in HL2, and errors in localizing the L_Model_ and L_Patient_. First, the patient’s position in the OR (lateral decubitus position) was different from the CT images (supine position), and the patient’s body was deformed with this change in position. Second, the surgeons were confronted with the impairment of depth perception caused by the HL2’s display system, where the displays are at a fixed distance from the eyes, while the distance from the eyes to the patient model varies [13]. This impairment was exacerbated by the SSRF setting, which requires the surgeon to locate the fractures inside the patient’s body [8]. Finally, the phantom study used explicit indicators (i.e., metal washers) for L_Model_ and L_Patient_, which reduced potential user error in L_Model_ and L_Patient_ localization. However, these indicators could not be used in the preclinical and clinical studies due to limitations in modifying the standard scanning protocol. Nevertheless, S1 and S2 commented that their perceived accuracy was considered useful for incision design. This was also validated in the clinical study where S2 was able to palpate the ribs based on their representative visualization in RibMR.

In the clinical study, RibMR identified 22 rib fractures, 8 more than US. RibMR demonstrated an 88% localization rate (22 out of 25 rib fractures), surpassing US by 22% points (56% localization rate; 14 out of 25 rib fractures). Additionally, RibMR successfully located subscapular rib fractures in both SSRFs, which were not located using the US. RibMR also demonstrated more consistent accuracy (1.38 ± 0.96 cm) for the 13 rib fractures identified by both RibMR and US with better average accuracy than US (3.46 ± 5.46 cm).

RibMR took an average of 4.42 ± 0.98 min to locate the simulated rib fractures in the phantom study, while the preclinical and clinical studies took longer by 3.61 and 4.34 min, respectively. We attribute these longer times to the increased difficulty of locating the L_Model_ and L_Patient_ in human patients. For example, in the preclinical study, surgeons took an average of 3.64 ± 3.18 min to locate the L_Model_, compared to an average of 0.53 ± 0.20 min in the phantom study. In the clinical study, it took an average of 4.59 ± 1.05 min to locate L_Patient_, compared to an average of 1.34 ± 0.32 min in the phantom study. However, RibMR took less time to locate rib fractures than US by 1.23 min in the clinical study. It is worth noting that US in our clinical study was faster than that reported by Huerly et al. [5], who took an average of 13 min to locate 14 fractures in 10 patients. We attribute RibMR’s faster speed to its wider FoV, which saves time in scanning each rib along its longitudinal axis. In addition, we believe that surgeon time can be reduced by offloading the model-patient alignment procedure to technicians or nurses, which took an average of 6.33 ± 0.79 min in the clinical study.

The surgeons identified and located rib fractures satisfactorily from the aligned patient model. S2 was satisfied with the rib fracture annotation model which clearly showed where the fractures were located. However, the surgeons highlighted the model’s depth perception and incomplete representation of the ribs as areas most in need of improvement. In the current implementation, the depth perception impairment was temporarily resolved using HL2’s finger-tracking feature. Based on the position of the finger in the HL2 displays and on the patient’s body, the perceived depth of the aligned patient model was aligned with the patient. In the second SSRF, S2 commented that this helped to mitigate the impairment and resulted in the best average accuracy of 0.71 ± 1.89 cm on human patients. Rib fracture annotation models were used as the primary determinant of rib fractures to address the concern of partial rib loss.

RibMR is designed with a simplified setup on mobile hardware, and the system could be set up in any OR at short notice. The system worked with the standard CT scanning protocol and was seamlessly integrated into existing clinical workflows. While RibMR has received positive feedback from all participating surgeons, maximizing the benefits of RibMR may require prior experience with MR or in-depth training before its use. For example, in the preclinical study, S3 had significantly lower rib fracture localization accuracy (*p* < 0.05, as determined by one-way ANOVA and Tukey HSD tests) than S1 and S2. We presume this is due to S3’s lack of experience with MR.

We compared our RibMR results with published work using MR in different surgical applications. In the phantom study, our accuracy (0.38 ± 0.21 cm) was similar to other applications, such as pedicle screw placement [11] and orthopedic surgery [9] where the authors reported an average accuracy of 0.25 ± 0.04 and 1.09 ± 0.04 cm, respectively. In the clinical study comparisons, our results (1.47 ± 1.33 cm) were less accurate by 1.03 cm compared to a neurosurgery study [10] (0.44 ± 0.25 cm), and also by 0.17 cm compared to a parotid gland tumor surgery [14] (1.30 ± 0.49 cm). We attribute these reductions to the complexity of rib fracture localization and the unique challenges identified for SSRF. For example, none of the compared systems accounted for the body deformation seen in our patient cases. Another example is the limited ability to modify the standard CT scanning protocol in SSRF, which prevented the elimination of user error in L_Model_ and L_Patient_ localization by attaching fiducial markers prior to the medical scan.

This study focused on the technical development, implementation, and comparative evaluation of RibMR to competing technologies. A clinical pilot study on the feasibility of our RibMR for SSRF with additional patient studies will be published as a separate work.

### Limitations and Future Work

The successful application of RibMR in four patients (2 patients × 2 studies) with different body habitus and fracture locations demonstrated its feasibility. However, the clinical study involved a limited number of patients and surgeons. Future work will include more extensive evaluations with additional patients and surgeons.

A limitation of this study is the use of 2D U-Net models for the segmentation of the bones. While 2D U-Net is widely adopted in the medical image analysis community and has demonstrated effectiveness in medical image segmentation tasks [37], there are more advanced models, such as nnU-Net [38] and 3D U-Net [39], which could offer improved performance (see Appendix). However, the 3D patient models generated from our 2D U-Net models were sufficient for the clinical requirements of our preclinical and clinical studies. Future work will focus on integrating more advanced segmentation models to further enhance the accuracy and robustness of the segmentation process.

During the clinical study, misalignment of the patient’s arms in the 3D patient model occurred due to differing patient poses in the OR, as discussed in the introduction, and the rigid nature of the model. However, this misalignment did not impact the localization of rib fractures during the clinical study, as appropriate alignment was achieved for the torso. In future work, we aim to incorporate non-rigid alignment using parametric human models (e.g., Skinned Multi-Person Linear model (SMPL) [40]) to ensure comprehensive alignment between the 3D model and the patient in the OR.

Our RibMR has demonstrated the ability to identify rib fractures more accurately which can lead to smaller incisions, and has also shown the ability to identify additional fractures that were not identifiable with US. Future studies will measure the clinical benefits of these RibMR benefits in terms of patient outcomes, such as better pain control management and postoperative recovery.

A key limitation of the current RibMR implementation was the depth perception issue, which prevented accurate visualization of the patient model. We anticipate that improved hardware in future generations of MR will help avoid this limitation and allow for more accurate perception.

The implementation of a markerless registration technique, which uses 3D surface information from the skin model and the patient’s body for the model-patient alignment, can potentially reduce user error in the model-patient alignment procedure. However, existing techniques lack the precision required for SSRF, i.e., Tu et al. [41] found that even the latest techniques resulted in an offset of up to 8.63 cm on a rigid human phantom. We suggest that appropriate handling of patient body deformation will be a critical factor in achieving the acceptable accuracy required for SSRF. Future research aims to develop a markerless registration technique that addresses this deformation issue and evaluates its feasibility in SSRF.

## Conclusion

We introduced RibMR – an MR-based visualization system for SSRF that projects a 3D patient model extracted from CT images onto the patient’s body. RibMR demonstrated its effectiveness for rib fracture localization in a controlled phantom study as well as in preclinical and clinical studies. Due to the limitations of CT images in intraoperative rib fracture localization without palpation or US, we assessed RibMR against US for accuracy, speed, and localization rate. Improved accuracy, speed, and higher localization rate of rib fracture localization were observed with RibMR compared to US practice with a greater number of fracture localizations and the ability to mark fractures occluded by other structures. However, our findings suggest the need for a more comprehensive model-patient alignment technique to fully realize the potential of RibMR in SSRF.

## Supplementary Material

Below is the link to the electronic supplementary material.

Supplementary Material 1 (PDF 463 KB)

Supplementary Material 2 (MP4 24.3 MB)

## References

[1] J. T. Prins, M. M. Wijffels, and F. M. Pieracci, “What is the optimal timing to perform surgical stabilization of rib fractures?,” *J. Thorac. Dis.*, vol. 13, no. Suppl 1, p. S13, (2021). 10.21037/jtd-21-649 PMC8371546 34447588

[2] Q. Zhang, L. Song, S. Ning, H. Xie, N. Li, and Y. Wang, "Recent advances in rib fracture fixation," *J. Thorac. Dis.*, vol. 11, no. Suppl 8, p. S1070, (2019). 10.21037/jtd.2019.04.99 PMC6545518 31205764

[3] T. J. Martin, J. Cao, E. Benoit, and T. Kheirbek, "Optimizing surgical stabilization of rib fractures using intraoperative ultrasound localization," *J. Trauma Acute Care Surg*., vol. 91, no. 2, pp. 369–374, (2021). 10.1097/TA.0000000000003262 33938512

[4] F. Turk, A. B. Kurt, and S. Saglam,"Evaluation by ultrasound of traumatic rib fractures missed byradiography," *Emerg. Radiol*., vol.17, no. 6, pp. 473-477, (2010). 10.1007/s10140-010-0892-9 20652719

[5] M. E. Hurley, G. D. Keye, and S. Hamilton, "Isultrasound really helpful in the detection of rib fractures?," *Injury*, vol. 35, no. 6, pp. 562-566, (2004). 10.1016/S0020-1383(03)00263-8 15135274

[6] S. Mariacher-Gehler and B. Michel, "Sonography: a simple way to visualize rib fractures," *AJR. Am. J. Roentgenol.*, vol. 163, no. 5, pp. 1268–1268, (1994) 10.2214/ajr.163.5.7976923 7976923

[7] J. P. Schots*et al*., "Addition of video-assisted thoracoscopic surgery to thetreatment of flail chest," *Ann.Thorac. Surg*., vol. 103, no. 3, pp. 940-944, (2017). 10.1016/j.athoracsur.2016.09.036 27939010

[8] C. Gsaxner *et al.*, “The HoloLens in medicine: A systematic review and taxonomy,” *Med. Image Anal*., p. 102757, (2023). 10.1016/j.media.2023.102757 36706637

[9] A. Teatini, R. P. Kumar, O. J. Elle, and O. Wiig, "Mixed reality as a novel tool for diagnostic and surgical navigation in orthopaedics," I*nt. J. Comput. Assist. Radiol. Surg.*, vol. 16, pp. 407–414, (2021). 10.1007/s11548-020-02302-z PMC7946663 33555563

[10] T. P. van Doormaal, J. A. van Doormaal, and T. Mensink, "Clinical accuracy of holographic navigation using point-based registration on augmented-reality glasses," *Oper. Neurosurg.*, vol. 17, no. 6, p. 588, (2019). 10.1093/ons/opz094 PMC6995446 31081883

[11] J. T. Gibby, S. A. Swenson, S. Cvetko, R. Rao, and R. Javan, “Head-mounted display augmented reality to guide pedicle screw placement utilizing computed tomography,” *Int. J. Comput. Assist. Radiol. Surg*., pp. 1–11, (2018). 10.1007/s11548-018-1814-7 29934792

[12] R. Moreta‐Martinez, D. García‐Mato, M. García‐Sevilla, R. Pérez‐Mañanes, J. Calvo‐Haro, and J. Pascau, "Augmented reality in computer‐assisted interventions based on patient‐specific 3D printed reference," *Healthc. Technol. Lett.*, vol. 5, no. 5, pp. 162–166, (2018). 10.1049/htl.2018.5072 PMC6222179 30464847

[13] C. Gsaxner et al., "Augmented reality for head and neck carcinoma imaging: Description and feasibility of an instant calibration, markerless approach," *Comput. Methods Programs Biomed*., vol. 200, p. 105854, (2021). 10.1016/j.cmpb.2020.105854 33261944

[14] C. Scherl et al., “Augmented reality with HoloLens® in parotid tumor surgery: a prospective feasibility study”, *ORL*, vol. 83, no. 6, pp. 439–448, (2021). 10.1159/000514640 33784686

[15] D. E. Meyer *et al.*, "Randomized controlled trial of surgical rib fixation to nonoperative management in severe chest wall injury," *Ann. Surg*., vol. 278, no. 3, pp. 357–365, (2023). 10.1097/SLA.0000000000005950 PMC10527348 37317861

[16] G. H. Golub and C. Reinsch, "Singular value decomposition and least squares solutions," in *Handbook for Automatic Computation: Volume II: Linear Algebra, *F. L. Bauer, A. S. Householder, F. W. J. Olver, H. Rutishauser, K. Samelson, and E. Stiefel Eds. Berlin, Heidelberg: Springer Berlin Heidelberg, (1971), pp. 134–151.

[17] O. Ronneberger, P. Fischer, and T. Brox, “U-net: Convolutional networks for biomedical image segmentation,” in *Medical Image Computing and Computer-Assisted Intervention – MICCAI*, (2015), pp. 234–241.

[18] M. E. Rayed, S. S. Islam, S. I. Niha, J. R. Jim, M. M. Kabir, and M. Mridha, “Deep learning for medical image segmentation: State-of-the-art advancements and challenges,”* Informatics in Medicine Unlocked*, p. 101504, (2024).

[19] J.-A. Pérez-Carrasco, B. Acha, C. Suárez-Mejías, J.-L. López-Guerra, and C. Serrano, “Joint segmentation of bones and muscles using an intensity and histogram-based energy minimization approach,” *Comput. Methods Programs Biomed*., vol. 156, pp. 85–95, (2018). 10.1016/j.cmpb.2017.12.027 29428079

[20] B. Rister, D. Yi, K. Shivakumar, T. Nobashi, and D. L. Rubin, "CT-ORG, a new dataset for multiple organ segmentation in computed tomography," *Scientific Data,* vol. 7, no. 1, p. 381, (2020). 10.1038/s41597-020-00715-8 PMC7658204 33177518

[21] W. E. Lorensen and H. E. Cline, "Marching cubes: A high resolution 3D surface construction algorithm," *ACM siggraph computer graphics*, vol. 21, no. 4, pp. 163–169, (1987).

[22] C. K. Reinbothe, T. Boubekeur, and M. Alexa, “Hybrid Ambient Occlusion,” *Eurographics*, vol. 5, 2009.

[23] M. Garland and P. S. Heckbert, "Surface simplification using quadric error metrics," in* Proceedings of the 24th annual conference on Computer graphics and interactive techniques*, (1997), pp. 209–216.

[24] H. Kato and M. Billinghurst, "Marker tracking and hmd calibration for a video-based augmented reality conferencing system," in *IEEE **and ACM International Workshop on Augmented Reality*, (1999): IEEE, pp. 85–94.

[25] K. S. Arun, T. S. Huang, and S. D. Blostein, “Least-squares fitting of two 3-D point sets”, *ITPAM*, no. 5, pp. 698–700, (1987). 10.1109/tpami.1987.4767965 21869429

[26] M. Alexandre, “Pytorch-UNet”, https://github.com/milesial/Pytorch-UNet (accessed Aug., 2020).

[27] A. Paszke *et al.*, “Pytorch: An imperative style, high-performance deep learning library,” *Adv. Neural Inf. Process. Syst*., vol. 32, (2019).

[28] A. Fedorov et al., "3D Slicer as an image computing platform for the Quantitative Imaging Network," *Magn. Reson. Imaging*, vol. 30, no. 9, pp. 1323–1341, (2012). 10.1016/j.mri.2012.05.001 PMC3466397 22770690

[29] P. Cignoni, M. Callieri, M. Corsini, M. Dellepiane, F. Ganovelli, and G. Ranzuglia, “Meshlab: an open-source mesh processing tool,” in *Eurographics*, (2008), vol. 2008: Salerno, Italy, pp. 129–136.

[30] Unity. “Unity.” https://unity.com/ (accessed Aug., 2020).

[31] Dummiesman. "Runtime OBJ Importer." https://assetstore.unity.com/packages/tools/modeling/runtime-obj-importer-49547 (accessed Aug., 2020).

[32] Microsoft. “MixedRealityToolkit-Unity.” https://github.com/microsoft/MixedRealityToolkit-Unity (accessed Aug., 2020).

[33] L. Qian. “HoloLens with ARToolKit v0.3.” https://github.com/qian256/HoloLensARToolKit (accessed Aug., 2020).

[34] Mathnet. "Math.NET Numerics." https://github.com/mathnet/mathnet-numerics (accessed Aug., 2020).

[35] C. Gsaxner, J. Li, A. Pepe, Y. Jin, J. Kleesiek, D.Schmalstieg, & J. Egger, “The HoloLens in medicine: A systematic review andtaxonomy”. *Med. Image Anal*., 85, 102757, (2023). 10.1016/j.media.2023.102757 36706637

[36] H. Scheffe, *The analysis of variance*, vol. 72,New York: John Wiley and Sons, (1999).

[37] M. Alexandre, “Pytorch-UNet”, https://github.com/milesial/Pytorch-UNet (accessed Aug., 2020). 31144149 10.1007/s10278-019-00227-x PMC6646484

[38] F. Isensee, P. F. Jaeger, S. A. Kohl, J. Petersen,and K. H. Maier-Hein, "nnU-Net: a self-configuring method for deeplearning-based biomedical image segmentation," *Nat. Methods*, vol.18, no. 2, pp. 203-211, (2021). 10.1038/s41592-020-01008-z 33288961

[39] Ö. Çiçek, A. Abdulkadir, S. S. Lienkamp, T. Brox, and O. Ronneberger, "3D U-Net: learning dense volumetric segmentation from sparse annotation," in* Medical Image Computing and Computer-Assisted Intervention*, (2016), Springer, pp. 424–432.

[40] M. Loper, N. Mahmood, J.Romero, G. Pons-Moll, and M. J. Black, "SMPL: A skinned multi-personlinear model," *ACM transactions on graphics (TOG)*, vol. 34, no. 6,pp. 1-16, (2015).

[41] M. Tu, H. Jung, A. Moghadam, J. Raythatha, J. Hsu, and J. Kim, "Exploring the Performance of Geometry-Based Markerless Registration in a Simulated Surgical Environment: A Comparative Study of Registration Algorithms in Medical Augmented Reality," in *International Conference of the IEEE Engineering in Medicine and Biology Society*, (2023): IEEE. 10.1109/EMBC40787.2023.10341197 38083251

