# Supplementary material for: RibMR – A Mixed Reality Visualization System for Rib Fracture Localization in Surgical Stabilization of Rib Fractures: Phantom, Preclinical, and Clinical Studies
Source: J Imaging Inform Med. 2024 Dec 20;38(5):3279–93. doi: 10.1007/s10278-024-01332-2 (PMC12572520; doi:10.1007/s10278-024-01332-2)
Supplement: Supplementary file 1 — Supplementary Material 1 (PDF 463 KB) [file 10278_2024_1332_MOESM1_ESM.pdf]

RibMR – A Mixed Reality Visualization System for Rib Fracture Localization in Surgical Stabilization of Rib Fractures: Phantom, Preclinical, and Clinical Studies – Appendix

Hoijoon Jung[1], Jineel Raythatha[2,3], Alireza Moghadam[2,3], Ge Jin[1], Jiawei Mao[1], Jeremy Hsu[2,3], and Jinman Kim[1*]

[*] Corresponding author (e-mail: jinman.kim@sydney.edu.au)

[1] Biomedical Data Analysis and Visualisation (BDAV) Lab, School of Computer Science, The University of Sydney, Camperdown, NSW, Australia, 2050

[2] Trauma Service, Westmead Hospital, Westmead, NSW, Australia, 2145

[3] Department of Surgery, Faculty of Medicine and Health, The University of Sydney, Camperdown, NSW, Australia, 2050

1 Preprocessing bone and body segmentation performance

1.1 Segmentation performance evaluation setting

The segmentation performance was evaluated for both body and bones. Rib fracture segmentation was not assessed, as these were manually segmented by surgeons. The The CT images across various studies (phantom, preclinical, and clinical) were also not assessed as they did not have the ground truth data. Instead, we used the public datasets of AutoPET [3], Usevilla Bone and Muscle [4], and CT-ORG [5], which included ground truth labels, to evaluate the body and bone segmentations. Performance metrics included Dice coefficient (DSC), Jaccard index, volume similarity, balanced average (BA) Hausdorff distance [1], and mean surface distance, following methodologies by Saha et al. [2].

Body segmentation (excluding respiratory structures) was evaluated using 20 CT studies randomly selected from the AutoPET dataset, with ground truth segmentations from the Atlas dataset [6]. The average size of these CT studies was 512×512×370.4 voxels. As the body was segmented using a thresholding technique without the need for training, the evaluation was simplified with a limited number of CT studies.

Bone segmentation was assessed via five-fold cross-validation using Usevilla Bone and Muscle [4] (27 CT studies with an average size of 512×512×10 voxels) and CT-ORG [5] datasets (140 CT studies with an average size of 512×512×453.6 voxels). Two 2D U-Net models [7] were separate trained on each dataset, with training ceasing after 200 epochs. Training was initialized with a learning rate of 0.0001 and the Root Mean Square Propagation (RMSprop) optimizer with a momentum factor of 0.9. Validation sets from each fold and dataset were processed through both models, and the resulting segmentations were aggregated as detailed in Section 2.1.1, "Segmentation of body, bones, and rib fractures," of the manuscript.

To benchmark our bone segmentation results, we compared the performance metrics of our technique with those of 3D U-Net [8], nnU-Net with the 3D full-resolution backbone [9], and TotalSegmentator [10]. The 3D U-Net was implemented using the Monai framework [11], based on the framework's example code [12], with the Dice loss function applied. Both 3D U-Net and nnU-Net were trained on the same datasets for 200 epochs. The 3D U-Net was initialized with a learning rate of 0.001 using the Adam optimizer, while nnU-Net used its default automatic settings. 3D U-Net and nnU-Net were evaluated through five-fold cross-validation. Unlike our technique, the validation sets were segmented by the corresponding 3D U-Net and nnU-Net models without aggregation of the resulting segmentations. TotalSegmentator, being a pre-trained tool for anatomical segmentation from CT images, was used without additional training on both datasets.

1.2 Result

Table 1 provides the segmentation performance metrics for the body and the bones. One CT study from the CT-ORG dataset was excluded due to a missing ground truth bone label. Additionally, two CT studies from the Usevilla Bone and Muscle dataset were excluded because they contained only a single leg.

Table 1 Segmentation performance for the body and the bones, measured using DSC, Jaccard index, volume similarity, BA Hausdroff distance (dist.), and mean surface dist.

Structures	DSC	Jaccard index	Volume similarity	BA Hausdroff dist. (mm)	Mean surface dist. (mm)
Body	0.986 ± 0.005	0.972 ± 0.009	0.026 ± 0.010	6.086 ± 5.193	5.402 ± 4.263
Bones	0.748 ± 0.123	0.612 ± 0.146	0.258 ± 0.230	18.931 ± 28.570	12.563 ± 12.713

Fig. 9 shows the visualizations of the body and the bone segmentation with contours derived from the segmentation. The predicted body segmentation displayed good overall overlap (green areas) with the ground truth, as indicated by the performance metrics. However, the technique over-segmented the bronchus (light blue arrows) and a small part of the CT scanner bed (orange arrows). These segmentation results were corrected for errors from the bronchus, other internal structures, and artifacts by the development team and surgeon during manual refinement.

In bone segmentation, our technique successfully segmented ribs (yellow arrows). There were over-segmentations of the aorta (purple arrows), an issue also seen in the patient's CT images. The surgeon was able to distinguish bones from the aorta during the clinical study.

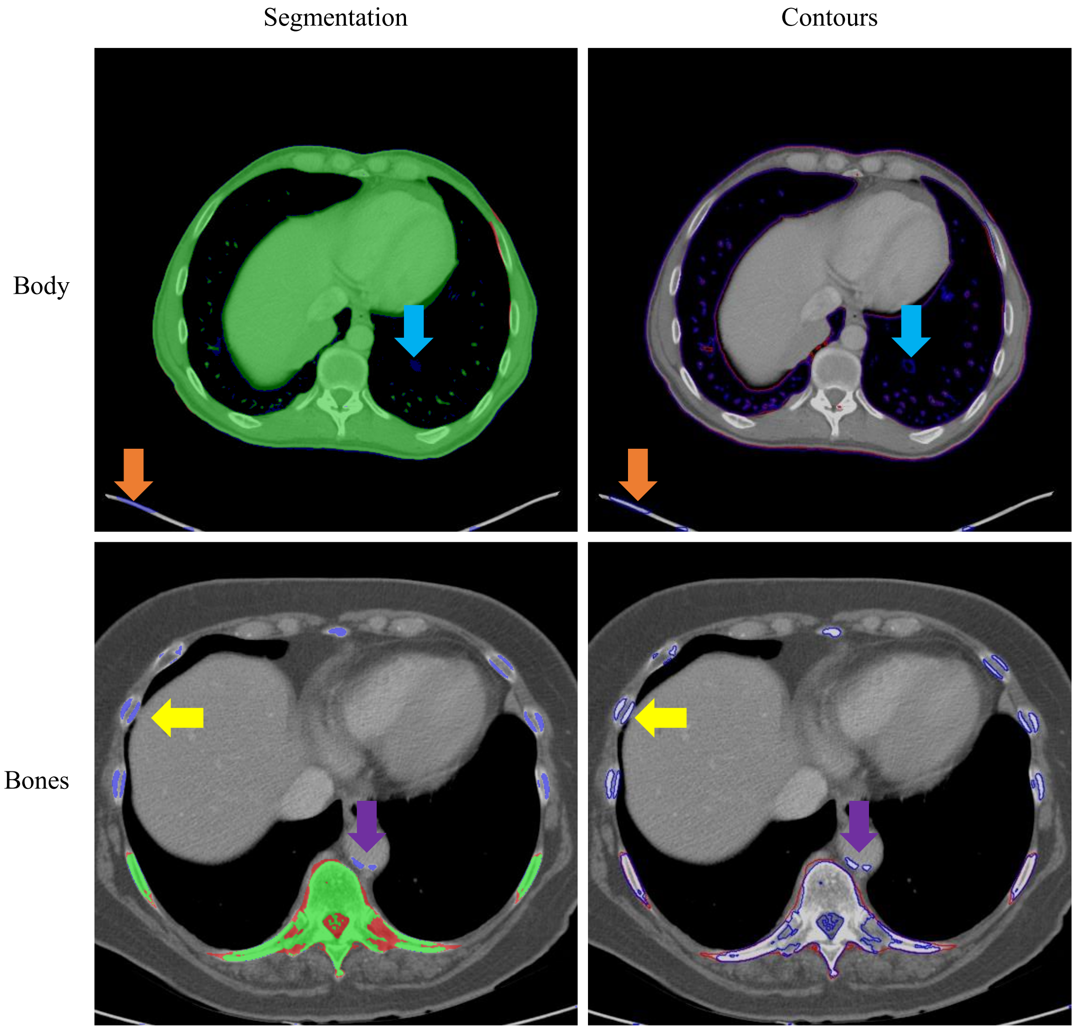

Fig. 9 Example visualizations of body and bone segmentation with contours: red indicates ground truth, blue indicates prediction, and green (segmentation only) indicates the overlap between ground truth and prediction.

Table 2 Segmentation performance comparison between our technique, 3D U-Net, nnU-Net, and TotalSegmentator for the bone segmentation. Metrics include DSC, Jaccard Index, Volume Similarity, BA Hausdorff dist., and Mean surface dist. The best of each metric is highlighted in bold. *One CT study from the CT-ORG was excluded due to empty resulting segmentation.

Techniques	DSC	Jaccard index	Volume similarity	BA Hausdroff dist. (mm)	Mean surface dist. (mm)
Ours	0.748 ± 0.123	0.612 ± 0.146	0.258 ± 0.230	18.931 ± 28.570	12.563 ± 12.713
3D U-Net	0.804 ± 0.155	0.695 ± 0.177	0.200 ± 0.314	16.882 ± 51.458	8.908 ± 16.173
nnU-Net	**0.907 ± 0.060**	**0.834 ± 0.089**	**0.070 ± 0.078**	**4.107 ± 4.361**	**3.897 ± 3.773**
TotalSegmentator*	0.674 ± 0.248	0.550 ± 0.227	0.292 ± 0.393	12.615 ± 18.072	12.681 ± 16.495

Table 2 presents a comparison of segmentation performance between our technique, 3D U-Net, nnU-Net, and TotalSegmentator. Three CT studies excluded from Table 1 were also omitted from this performance comparison. Additionally, TotalSegmentator failed to segment bones in one CT study from the CT-ORG dataset, resulting in an empty segmentation. This case was excluded from the results of TotalSegmentator. Our method achieved a DSC of 0.748 ± 0.123, outperforming TotalSegmentator (0.674 ± 0.248), though slightly lower than 3D U-Net (0.804 ± 0.155). nnU-Net demonstrated the highest DSC at 0.907 ± 0.060. A similar trend was observed for the Jaccard index, volume similarity, and mean surface distance, where our technique showed better performance than TotalSegmentator. However, for BA Hausdorff distance, our technique performed worse than the others.

During the preprocessing of the CT-ORG dataset, we identified potential errors such as missing ground truth bone labels, incorrect ground truth labels, e.g., low-quality rib segmentations. It is expected that with improved ground truth labels, the performance of our bone segmentation technique will improve. In this study, the automatic bone segmentation primarily served as a guide for surgeons to confirm bony landmarks on the patient's skin model and to locate rib fractures with the rib fracture annotations. Throughout the preclinical and clinical studies, the 3D patient models constructed from segmentations using our techniques were adequate for our clinical purpose.

2 Experimental settings

2.1 Phantom study

2.1.1 Locations of 32 metal washers

Fig. 10 shows the patient phantom with 32 metal washers. Eight washers were placed at 10 candidate rib landmarks, excluding the Xiphi and T1 vertebrae. The remaining 24 washers were placed along the estimated locations of the 7th through 10th ribs in six directions (3 positions (anterior, posterior, and lateral) × 2 sides (left and right)) to represent simulated rib fractures located.

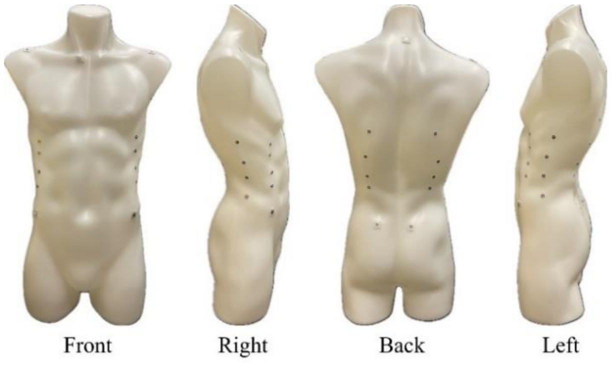

Fig. 10 The patient phantom in multiple angles with the 32 metal washers

2.1.2 Locations of five $L_{Model}/L_{Patient}$ and 16 simulated rib fractures

Table 3 shows the five simulated $L_{Model}/L_{Patient}$ pairs, and 16 selected simulated rib fractures were used to align the model and measure the accuracy and speed of RibMR.

Table 3 The five simulated $L_{Model}/L_{Patient}$ and 16 simulated rib fractures used in each phantom position.

Phantom position	Five simulated $L_{Model}/L_{Patient}$	16 simulated rib fractures
Supine	Suprasternal notch, left and right AC joints, left and right ASIS	Simulated fractures on anterior and lateral positions of left and right
Right lateral decubitus	Suprasternal notch, left AC joint, C7 vertebrae, left ASIS and left PSIS	Simulated fractures on left and right posterior, left anterior and left lateral positions
Left lateral decubitus	Suprasternal notch, right AC joint, C7 vertebrae, right ASIS, right PSIS	Simulated fractures on left and right posterior, right anterior, and right lateral positions

2.1.3 Phantom with the aligned model

Fig. 11 shows the phantom in the right lateral decubitus position, aligned with its corresponding model.

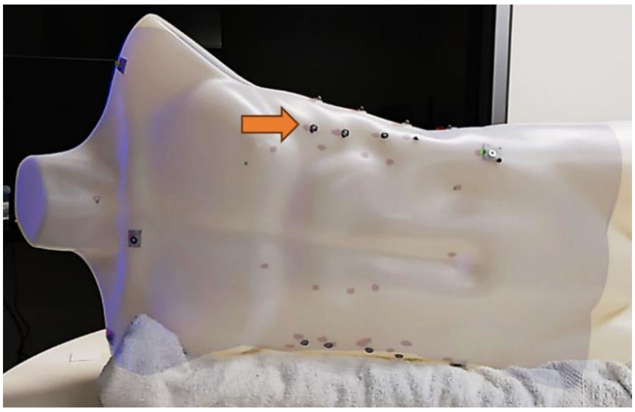

Fig. 11 The phantom in the right lateral decubitus position with its model aligned. The phantom skin model is shown in a semi-transparent blue and the rib fracture annotation model is displayed in semi-transparent red where the orange arrows point

2.2 Preclinical study

2.2.1 Patient demographics

Table 4 shows the demographics of the patients participated in the preclinical study.

Table 4 Demographics of patients in the preclinical study.

Patient	Gender	Height (cm)	Weight (kg)	BMI	Side of simulated fractures	Position in the study
P2	Male	172	77	26.03	Left	Right lateral decubitus
P3	Male	173	70	23.39	Front	Supine

2.2.2 Patient positions on the simulated operating table

Fig. 12 shows the positions of P3 and P2 on the simulated operating table.

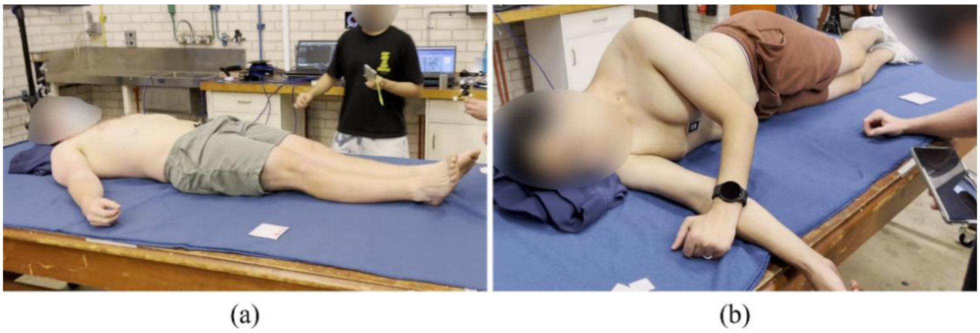

Fig. 12 Two healthy patients of the preclinical study in (a) supine (P3) and (b) right lateral decubitus positions (P2)

2.2.3 Locations of six simulated rib fractures

Table 5 shows the location of six simulated rib fractures for each patient in the preclinical study.

Table 5 The locations of six simulated rib fractures for each patient.

Patient (Position)	Locations of six simulated rib fractures
P3 (Supine)	Suprasternal notch, Xiphi, left and right AC joints, left and right 10th rib tips
P2 (Right lateral decubitus)	Suprasternal notch, Xiphi, left AC joint, C7 and T10 vertebrates, left scapular tip

2.3 Clinical study

2.3.1 Patient demographics

Table 6 shows the demographics of the patients in the clinical study.

Table 6 Demographics of patients in the clinical study.

SSRF	Gender	Height (cm)	Weight (kg)	BMI	Side of fractures	Position in OR
First SSRF	Male	180	80	24.69	Left	Right lateral decubitus
Second SSRF	Male	178	80	25.25	Right	Left lateral decubitus

2.3.2 Surgeons marking rib fractures using RibMR and ultrasound

Fig. 13 illustrates surgeons marking rib fractures on the patient during SSRF, using RibMR (Fig. 13a) and ultrasound (US) (Fig. 13b).

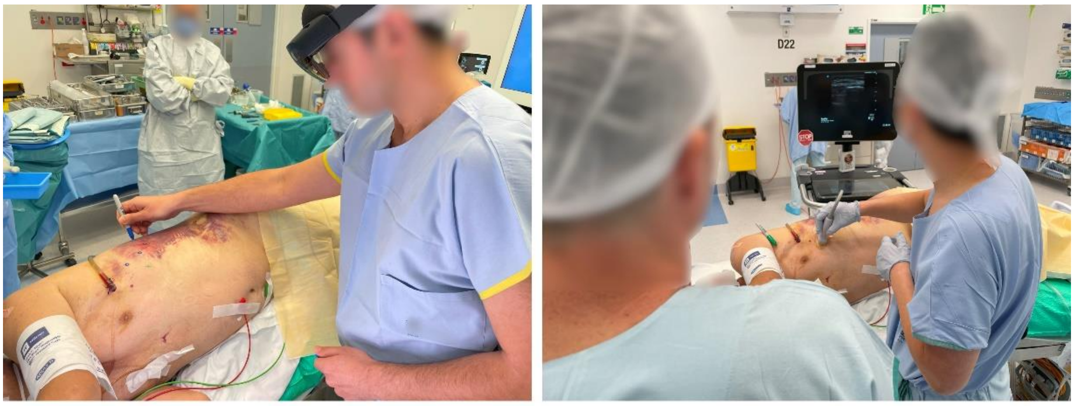

(a) (b)

Fig. 13 Rib fractures were marked using (a) RibMR and (b) US in the SSRF

2.3.3 Locations of L_{Model} and $L_{Patient}$

Table 7 shows the locations of L_{Model} and $L_{Patient}$ used in each SSRF in the clinical study.

Table 7 L_{Model} and $L_{Patient}$ used in SSRF.

SSRF	L_{Model} and $L_{Patient}$ used in SSRF
First SSRF	Suprasternal notch, left AC joint, C7 vertebrate, left ASIS, left PSIS
Second SSRF	Suprasternal notch, Xiphi, right AC joint, C7 vertebrate, right ASIS

3 Results

3.1 Rib fracture localization accuracy

3.1.1 Phantom study

RibMR showed an average accuracy of 0.25 ± 0.12 cm on the phantom in the supine position, 0.35 ± 0.15 cm in the right lateral decubitus position, and 0.55 ± 0.23 cm in the left lateral decubitus position. One-way ANOVA and Tukey HSD tests confirmed statistically significant differences ($p < 0.05$) in accuracy between the supine and left lateral decubitus positions and between the right and left lateral decubitus positions. However, there was no significant difference between the supine and right lateral decubitus positions (Tukey HSD $p > 0.05$).

3.1.2 Preclinical study

RibMR demonstrated an average accuracy of 2.94 ± 1.33 cm at P3 in the supine position and 4.72 ± 3.15 cm at P2 in the right lateral decubitus position. A two-sample t-test showed no statistical significance between the accuracies at P2 and P3 ($p > 0.05$).

S1 demonstrated an accuracy of 2.12 ± 0.79 cm at P3, which was significantly better (two-sample t-test $p < 0.05$) than S2's 3.77 ± 1.28 cm. S1 also showed significantly better accuracy (two-sample t-test $p < 0.05$) of 3.03 ± 1.58 cm at P2 than S3 (7.25 ± 3.37 cm). However, a two-sample t-test could not find a significant difference ($p > 0.05$) between S1's localization accuracies at P2 and P3.

3.1.3 Clinical study

RibMR yielded an average accuracy of 2.00 ± 0.00 cm in the first SSRF and 0.71 ± 1.89 cm in the second SSRF. A two-sample t-test showed no statistical significance between the accuracies of the first and second SSRFs.

In the first SSRF, 9 rib fractures were marked by both RibMR and US. RibMR showed an average accuracy of 2.00 ± 0.00 cm, while US showed a marginally lower accuracy (two-sample t-test $p >0.05$) of 5.00 ± 6.00 cm. In the second SSRF, 4 rib fractures were marked by both RibMR and US, and both methods showed an accuracy of 0.00 ± 0.00 cm.

3.2 Rib fracture localization times

In the phantom study, RibMR took 3.67 minutes on the phantom in the supine position, 5.53 minutes in the right lateral decubitus position, and 4.07 minutes in the left lateral decubitus position.

In the preclinical study, S1 and S2 took 4.42 and 13.03 minutes on P3 in the supine position, while S1 and S3 took 6.48 and 8.17 minutes on P2 in the right lateral decubitus position.

In the clinical study, RibMR took 9.22 and 8.30 minutes in the first and second SSRFs, respectively, while US took 14.50 and 5.47 minutes.

References

[1] O. U. Aydin et al., "On the usage of average Hausdorff distance for segmentation performance assessment: hidden error when used for ranking," *European radiology experimental*, vol. 5, pp. 1-7, (2021).

[2] M. Saha, J. W. Jung, S.-W. Lee, C. Lee, C. Lee, and M. M. Mille, "A deep learning segmentation method to assess dose to organs at risk during breast radiotherapy," *Physics and Imaging in Radiation Oncology*, vol. 28, p. 100520, (2023).

[3] S. Gatidis et al., "A whole-body FDG-PET/CT dataset with manually annotated tumor lesions," *Scientific Data*, vol. 9, no. 1, p. 601, (2022).

[4] J.-A. Pérez-Carrasco, B. Acha, C. Suárez-Mejías, J.-L. López-Guerra, and C. Serrano, "Joint segmentation of bones and muscles using an intensity and histogram-based energy minimization approach," *Comput. Methods Programs Biomed.*, vol. 156, pp. 85-95, (2018).

[5] B. Rister, D. Yi, K. Shivakumar, T. Nobashi, and D. L. Rubin, "CT-ORG, a new dataset for multiple organ segmentation in computed tomography," *Scientific Data*, vol. 7, no. 1, p. 381, (2020).

[6] A. Jaus et al., "Towards unifying anatomy segmentation: automated generation of a full-body CT dataset via knowledge aggregation and anatomical guidelines," *arXiv preprint arXiv*:2307.13375, (2023).
[7] O. Ronneberger, P. Fischer, and T. Brox, "U-net: Convolutional networks for biomedical image segmentation," in *Medical Image Computing and Computer-Assisted Intervention*, (2015), Springer, pp. 234-241.
[8] Ö. Çiçek, A. Abdulkadir, S. S. Lienkamp, T. Brox, and O. Ronneberger, "3D U-Net: learning dense volumetric segmentation from sparse annotation," in Medical Image Computing and Computer-Assisted Intervention, (2016), Springer, pp. 424-432.
[9] F. Isensee, P. F. Jaeger, S. A. Kohl, J. Petersen, and K. H. Maier-Hein, "nnU-Net: a self-configuring method for deep learning-based biomedical image segmentation," *Nat. Methods*, vol. 18, no. 2, pp. 203-211, (2021).
[10] J. Wasserthal et al., "Totalsegmentator: Robust segmentation of 104 anatomic structures in ct images," *Radiol. Artif. Intell*., vol. 5, no. 5, (2023).
[11] M. J. Cardoso et al., "Monai: An open-source framework for deep learning in healthcare," *arXiv preprint arXiv:2211.02701*, (2022).
[12] Monai, “MONAI tutorials”, https://github.com/Project-MONAI/tutorials (accessed Aug., 2024).
